# The refugee post-migration stress scale (RPMS) – development and validation among refugees from Syria recently resettled in Sweden

**DOI:** 10.1186/s13031-019-0246-5

**Published:** 2020-01-06

**Authors:** Andreas Malm, Petter Tinghög, Jurgita Narusyte, Fredrik Saboonchi

**Affiliations:** 1 Department of Health Sciences, The Swedish Red Cross University College, PO Box 1059, 141 21 Huddinge, Sweden; 2 Department of Clinical Neuroscience, Karolinska Institutet, Division of Insurance Medicine, 171 77 Stockholm, Sweden; 3 The Swedish Red Cross Treatment Center for Persons Affected by War and Torture, PO Box 166, 201 21 Malmö, Sweden

**Keywords:** Post-migration stress, Refugee, Assessment, Scale development, Construct validity, Confirmatory factor analysis, Exploratory factor analysis, Mental health, Syria

## Abstract

**Background:**

Despite the growing recognition of the impact of post-resettlement factors on the mental health of refugees, a clear definition of the concept of post-migration stress, as well as an updated, valid instrument for assessing the construct, are still lacking. The aim of the current study was to develop and validate the Refugee Post-Migration Stress Scale (RPMS), a concise, multi-dimensional instrument for assessing post-migration stress among refugees.

**Results:**

Based on a review of previous research and observations from a refugee trauma clinic, a preliminary 24-item instrument was developed, covering seven hypothesized domains of post-migration stress: *perceived discrimination, lack of host country specific competences, material and economic strain, loss of home country, family and home country concerns, social strain,* and *family conflicts*.

In the context of a population-based survey of mental health among refugees from Syria recently resettled in Sweden (*n* = 1215), the factorial structure of the RPMS was investigated. Confirmatory Factor Analysis revealed slightly insufficient fit for the initial theorized multi-domain model. Exploratory Factor Analysis in four iterations resulted in the omission of three items and an adequate fit of a 7-factor model, corresponding to the seven hypothesized domains of post-migration stress. To assess concurrent validity, correlational analyses with measures of anxiety, depression, post-traumatic stress disorder (PTSD), and mental wellbeing were carried out. All domains of post-migration stress showed significant correlations with anxiety, depression, and PTSD scores, and significant negative correlations with mental wellbeing scores.

**Conclusions:**

The newly developed RPMS appears to be a valid instrument for assessing refugee post-migration stress. Our findings that post-migration stress primarily relating to social and economic factors seems to be associated with mental ill health among refugees is in line with previous research.

## Background

With an unprecedented 70.8 million forcibly displaced people worldwide, the global crisis for those who are forced to leave their homes as a result of conflict and persecution shows no signs of abating. Of the displaced, nearly 25.9 million have crossed a border into another country, hence making them refugees according to international law, while another 3.5 million are asylum seekers [1]. Refugees typically have experiences of war, persecution and loss, with potentially traumatic events occurring both in the country of origin and during migration [2]. While living conditions for those refugees who end up in neighboring countries are often harsh, resettling in a socially and culturally unfamiliar country far from home may pose significant challenges of other kinds, laying additional burden on individuals who are already exposed to numerous risk factors. Elevated prevalence rates for mental ill health in refugee groups compared to the general population have been shown in several studies [3, 4]. Although reported rates vary substantially between different studies and population, it remains clear that refugees are at risk for developing long-lasting psychological disorders, such as post-traumatic stress disorder (PTSD), anxiety and depression [5, 6].

Historically, the primary focus in research has been on pre-migratory and trauma-related risk factors and their impact on refugees’ mental health, but recent years has seen a shift of focus towards the health implications of the living conditions that refugees face after resettlement in the host country (for a comprehensive review, see Li et al. [7]). In the same vein, post-resettlement factors have even been suggested to be of potentially greater importance than pre-migratory conditions for refugees’ mental health [4, 8, 9]. However, despite the increased attention given to the impact of post-resettlement factors on the mental health of refugees, a clear definition of the concept of post-migration stress and a validated measure for assessing post-migration stress among refugees are still lacking.

Several different types of resettlement experiences have been shown to be associated with mental ill health among refugees and migrants. These experiences include perceived discrimination [10, 11], low host-country language skills [12], being separated from family [9, 13], uncertainty relating to asylum application [8, 14], financial difficulties and unemployment [12, 15], lack of private accommodation [4], social isolation [16], loss of status [17, 18], poor social support [14, 19], and conflicts between spouses or parents and children [20, 21].

In general, there is ample evidence that socioeconomic adversities and disadvantaged living conditions are linked to mental ill health [22, 23]. The account of poor socioeconomic living conditions as causal agents inserting significant impact on mental ill health has been outlined as the *social causation hypothesis* [24]. This causal link may be viewed as asserting an indirect impact, e.g., through the poverty-based lack of access to healthcare services [25]. Another plausible pathway is through the impact mediated by psychosocial stress generated by the poor socioeconomic conditions [26, 27]. The psychosocial stress pathway has specifically been used to explain ethnicity-based health disparities [28]. By incorporating facets such as perceived racism [29] and social status incongruency [30], the psychosocial stress pathway appears to be applicable to post-resettlement living conditions of refugees.

Despite important conceptual differences, and possibly due to the lack of a broadly accepted definition, terms such as *post-migration stress* [7], *post-migration living difficulties* [31] and *resettlement stressors* [17] may appear confusingly similar, implying a need for clarification. When attempting to disentangle the conceptual models of the post-resettlement hardship faced by refugees, an important conceptual distinction is that of *stressors* and *stress as an outcome*, i.e., distress, while *stress* refers to the entire process [32]. *Stressors* can be defined as exposures, changes, threats and conditions that precipitate *distress*. As such, stressors can be episodical, or reoccurring or chronic, in face of which stress can be viewed as the cognitive and emotional processes that lead to the appraisal of these events as harmful or threatening [33].

In applying this conceptual distinction to resettlement experiences, constructs such as post-migration living difficulties could be classified as reflecting *stressors*, whereas post-migration stress would denote the process of subjective appraisal of such events as stressful, harmful or threatening. The real-life application of such theoretical distinction is not always straightforward, as has been implied in the development of assessment of perceived stress [34], daily hassles [35], and life events [36]. The real-life overlaps of stressors, stress, and stress responses may be seen as indicating that the outlining and assessment of post-migration stress should both target the subjective appraisals, as well as linking these appraisals to conditions that are inherently stressful for refugees.

In light of the above, we propose a definition of the concept of *refugee post-migration stress* as the *subjective appraisal of reoccurring or persistent post-resettlement related living conditions as distressing*. The concept of refugee post-migration stress also implies appraisal of one’s *resources to deal with the demands from such living conditions as insufficient*. Furthermore, given the multiplicity of stressor domains and stressful experiences outlined by the previous literature, refugee post-migration stress may appropriately be viewed as *multi-dimensional*. Finally, the distressing nature of the refugee post-migration stress suggests expected associations with mental ill health.

Although measures for assessing post-migration stress and related concepts among refugees and immigrants exist, the primary focus in these appears to be on stressors (e.g., [17, 31]). With a strong emphasis on stressors, it may be construed as if situations, events, and living conditions were inherently stressful in themselves, regardless of how they are perceived and interpreted. This may be true in some, or perhaps even many instances, but is by no means true for all [37]. In addition to instruments focusing on stressors, there are other instruments that cover stressful experiences related to migration, although not specifically relating to refugee experiences (e.g., [38, 39]). In all, there appeared to be a need for an updated assessment instrument for refugee post-migration stress, that is anchored in a clear definition of the concept of post-migration stress, and that reflects the current post-resettlement living conditions of refugees in high income countries.

The aim of the current study is to develop and validate a self-assessment instrument for evaluation of post-migration stress among refugees from Syria.

## Methods and materials

**Fig. 1:**
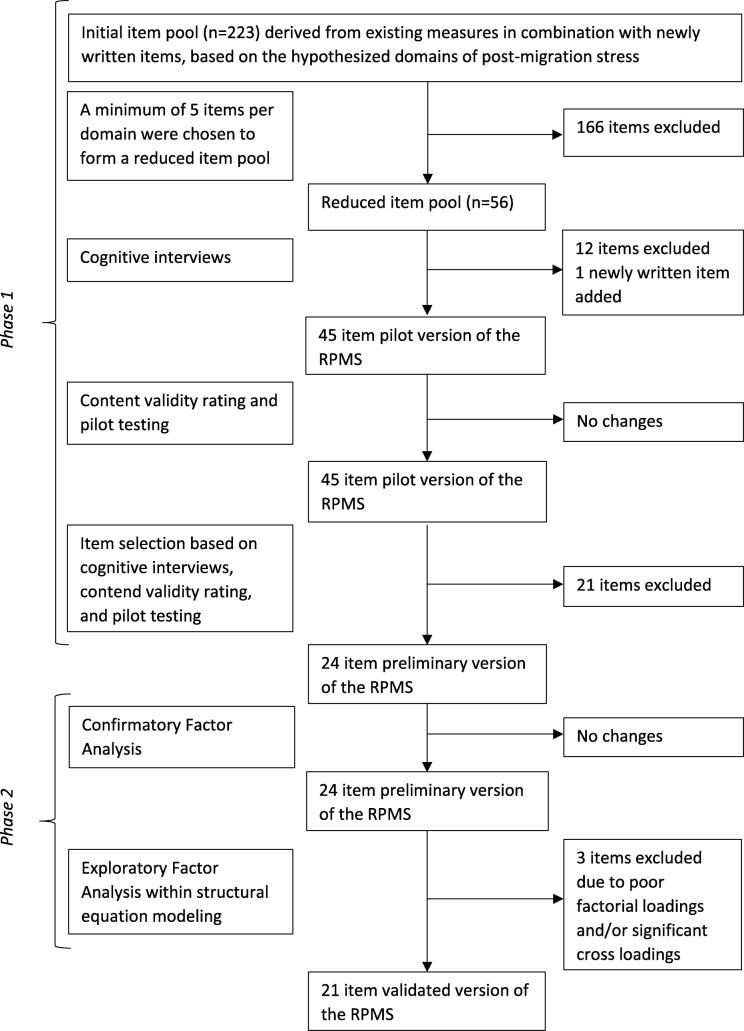
Development process for the Refugee Post-Migration Stress Scale (RPMS)

The development of the Refugee Post-Migration Stress Scale (RPMS) was carried out in two consecutive phases. The first phase involved construction of the new instrument, including pretesting and content validation. In the second phase, validity of the construct was assessed using confirmatory and exploratory factor analyses. The development process for the RPMS is shown in Fig. 1.

The development of the instrument was based on the recommendations by Clark and Watson [40] and Simms [41] and the concept of construct validity [42]. The same approach had previously been adopted in scale development work by our research group [43].

### Phase 1: instrument development

#### Conceptualization of constructs and item pool generation

In an iterative process, the conceptualization of the target constructs and the initial item pool were developed simultaneously.

A review of existing literature on post-migration stress and related constructs was conducted in search for commonly reported stressful experiences faced by refugees in post-resettlement contexts. To provide an exhaustive range, clinical observations regarding stressful experiences reported by refugee patients at the Swedish Red Cross Treatment Center for Persons Affected by War and Torture (RCC, where the first author is employed), not covered by the empirical studies were also added. Expert panel discussions with psychosocial professionals originating from Syria resulted in the inclusion of additional types of stressful experiences. The list of experiences was used to formulate domains of post-migration stress, and to encircle items for each domain.

An initial item pool of 223 items was generated by combining items from instruments identified in the literature review with newly written items based on the list of stressful experiences. These newly written items were formulated by the research group in the cases where available items did not sufficiently cover all relevant aspects of the hypothesized domains of post-migration stress. Where needed, the wording of items from other instruments were revised. By excluding redundant items from the initial item pool, a reduced item pool was reached.

#### Translation and back-translation

Items from the reduced item pool were translated into standard Arabic, using the procedure of translation and back-translation with independent translators. The translations were also discussed in a group of bilingual psychosocial professionals, and small revisions in the phrasing of items were made.

#### Cognitive interviews

To improve usability and reduce risk of measurement error, a cognitive interview procedure for pretesting the items of the instrument was adopted [44]. Cognitive interviews may inform the researcher on how respondents perceive and interpret the items of the questionnaire and may also provide information on problems that may arise trying to answer the questions. Cognitive interviews are of particular use for questions with sensitive or intrusive content, and when memory retrieval is required [45]. A convenience sample of *n* = 7 patients with Arabic as their mother tongue were recruited at the RCC. The cognitive interviews were conducted by the first author together with an experienced interpreter employed at RCC. A combination of two cognitive techniques, think-aloud protocol and probing, was used. The respondent was instructed to say out loud all that came into mind while reading and answering each item. When the respondent gave little or no information regarding a particular item, probes were used. For each item, reported difficulties were coded as being related to comprehension, memory retrieval, judgement or response formatting [44].

#### Content validity rating

To assess whether the included items adequately represented the hypothesized domains of the instrument, content validity rating was carried out [46]. For the content validity rating, clinicians at the RCC with extensive experience from work with refugees were recruited by email. Participants were asked to rate the relevance of each item in relation to its domain, and for each domain the rationale was provided. Domains and rationales are presented in Table 1.

**Table 1:** Hypothesized domains of post-migration stress, domain rationales, and sample items for each domain

Domain	Rationale	Sample items
Perceived discrimination	Self-experienced unfair verbal and non-verbal treatment, interpreted as being performed on the basis of prejudice, intentionally or unintentionally	“Feeling disrespected due to my national background”
Lack of host country specific competences	Individuals’ lack of skills that are instrumental for dealing adequately with and understanding situations, events and procedures that are fairly common in the new society	“Difficulties understanding documents and forms from authorities”
Material and economic strain	Material and economic hardship, that constitutes a threat against one’s integrity, independence, dignity and wellbeing in the host country	“Worry about unstable financial situation”
Loss of home country	A longing for a lifestyle and ways of interaction associated with the metaphorical home (or *heimat*), that was lost through (forced) migration and exile	“Longing for my home country”
Family and home country concerns	Distress resulting from the impact of past or present conflicts in the home country, and from the consequences these conflicts have on the individual’s family members and loved ones	“Worry about family members that I am separated from”
Social strain	Social hardship primarily associated with migration to and settlement in the host country society	“Frustration due to loss of status in the Swedish society”
Family conflicts	Conflicts with significant others that cause tension and/or unwanted emotional responses	“Distressing conflicts in my family”

Content validity rating was done on a 4-point scale. Responses were dichotomized (1 or 2 indicating *not relevant*, 3 or 4 indicating *relevant*). Content validity index for each item (I-CVI) was calculated. When using 6 or more raters, I-CVI should be at least 0.78 [47]. I-CVIs were used to calculate scale-level content validity index (S-CVI). S-CVI is suggested to be no lower than 0.9, although recommendations vary [48].

#### Pilot testing

Pilot testing, using the pilot version of the RPMS, was conducted on four different occasions. On the first two occasions, adult refugee students in a Swedish-for-immigrants program were invited to participate in the study via teachers from their language classes. On the third and fourth occasions, patients at the RCC participating in group activities led by clinicians that were not involved in the study, were invited to participate. The same oral and written information about the study was given to all participants.

#### Item selection for the preliminary instrument

The set of items from the pilot version of the RPMS was evaluated by the research group to reach a preliminary version of the instrument. The overall evaluation was based on usability of items, I-CVIs, preliminary statistical characteristics from pilot testing (mean and individual inter-item correlations, dispersion, floor and ceiling effects), theoretical considerations regarding item content, and adequate number of items per domain.

### Phase 2: construct validity

The second phase of the development involved assessment of construct validity by means of confirmatory and exploratory factor analyses, as well as correlational analyses for assessing concurrent validity.

#### Data collection and sample characteristics

The validity of the RPMS was assessed in the context of a population-based survey, in which a postal questionnaire including the RPMS was sent to a random sample of 4000 individuals from Syria aged 18–64 years who had been granted permanent residency in Sweden between 2011 and 2013. The sample was drawn from a known sample frame including all eligible participants.

#### Selected measures

Mental ill health and wellbeing was assessed using Hopkins Symptom Checklist 25 (HSCL-25), Harvard Trauma Questionnaire (HTQ), and WHO-5 Well-being index (WHO-5). All instruments have been used extensively in refugee populations.

HSCL-25 contains 10 items for measuring anxiety and 15 items for measuring depression. Items are answered by choosing one of four response alternatives, ranging from “not at all” (1) to “very much” (4) [49, 50].

HTQ part IV has a similar structure and response format as HSCL-25 and contains 16 items corresponding to the symptoms of the PTSD diagnosis [49].

WHO-5 was used to assess current mental wellbeing. The respondent answers five statements regarding how he or she has been feeling the last two weeks, by choosing from six response alternatives, ranging from “at no time” (0) to “all of the time” (5) [51].

#### Statistical analysis

At the item selection phase, each individual item from the pilot version of the RPMS was screened by inspecting the inter-item correlation coefficients, and the overall mean inter-item correlations. To investigate potential floor and ceiling effects, the dispersion of each item’s response categories was visually inspected.

Given the specific domains outlined by the conceptualization of post-migration stress, a 7-factor, theory-driven measurement model was initially examined by means of confirmatory factor analysis (CFA; 52) with maximum likelihood estimation and robust standard errors (MLR). Satorra–Bentler scaled chi-square test statistics were used to assess the overall model fit. Comparative Fit Index (CFI), root mean squared error of approximation (RMSEA), and standardized root mean square residual (SRMR) were also used to assess the goodness of fit of the model. The combination of fit indices minimizes the risk of rejection of well-fitting models, as chi-square test statistics are sensitive to large sample sizes. Non-significant chi-square test statistics, cutoff values close to 0.95 for CFI and 0.08 for SRMR, and values close to 0.06 for RMSEA indicate good fit between the model and the observed data [53].

Upon indications of lack of model fit, Modification Indices were inspected to examine theoretically justifiable modifications to the model. If no such modification was available, and/or in cases of several such model re-specifications necessary to improve the fit, exploratory factor analysis (EFA) with Geomin rotation within the framework of exploratory structural equation modelling (ESEM) was used to establish the dimensionality of the measurement. EFA within ESEM, which allows for non-zero cross-loadings and residual variances, was chosen, as further non-justifiable modifications would have increased the risk of overfitting the model [54]. Applied in this framework, the same fit indices as those for CFA are examined for each factorial solution providing means for non-arbitrary selection of the number of factors. Robust standard errors for factorial loadings are further provided to assess the significance of each item’s loadings. EFA is conducted in several iterations allowing for the identification and omitting of items with either substantive and significant cross-loadings or poor factor loadings.

Cronbach’s alpha was also assessed as a measure of internal consistency for each factor and the total instrument [55].

To assess the associations between post-migration stress and mental ill health, zero-order correlations between identified factors of the instrument and scores on HSCL-25, HTQ, and WHO-5 were calculated.

#### Statistical software

For the confirmatory and exploratory factor analyses, M*plus* V8.3 software was used. The correlational analyses were investigated using SPSS V24.0.

## Results

### Phase 1: instrument development

#### Conceptualization of constructs and item pool generation

The conceptualization process resulted in a 7-domain model of refugee post-migration stress. The domains and their conceptualizations are presented in Table 1. Four of the domains clearly related to host society stress (*perceived discrimination, lack of host country specific competences, material and economic strain,* and *social strain*), whereas three domains more closely related to family and home country stress (*loss of home country, family and home country concerns,* and *family conflicts*). For each domain, a minimum of five items were chosen from the initial item pool, resulting in a reduced item pool of 56 items.

#### Cognitive interviews

Five men and two women of varying ages (42–66, average 55 years) were recruited for the cognitive interviews. All were of Iraqi origin and indicated Arabic as their mother tongue, and all had refugee experiences. Time since arriving to Sweden ranged from 5 to 9 years (average 7 years).

The reduced item pool (*n* = 56) was used for the cognitive interviews. Results from the interviews indicated minor difficulties relating to comprehension of items. These difficulties were of two kinds: (1) difficulties understanding the literal meaning of specific words or phrases in the Arabic translation *(“behaviors”, “necessities”, “residential area”, “ordinary life activities”, “customs or values”, “everyday life”, “loss of status”*), and (2) uncertainty regarding what or who the item referred to *(“racist remarks, by who?”, “debts, to me or others?”, “communication, with whom?”*). For items containing the word *“difficulties”*, the Arabic phrase contained a double negation, which was grammatically correct, but nonetheless caused comprehension difficulties.

Interviewees reported difficulties relating to judgment for a few items. For one item, this was due to the double question in the item (*“discrimination in school or at work”*), and for two other items the concerns were related to experiences described in the item that the interviewee could not relate to (*“conflicts with spouse”* if one is unmarried, *“cannot practice my religion as I want”* if one does not confess to any religion). Another comment relating to the items in the *Family conflicts* domain concerned the word *“conflict”*, and how to judge what qualified as a conflict (which led to the revision of these items to “*distressing* conflicts …” ).

Despite the reported difficulties relating to comprehension and judgment, all items but one was answered by all interviewees (*“difficulties getting access to proper health care”*, which was excluded at this step). No concerns regarding retrieval or response formatting were detected.

The findings were discussed with experienced interpreters and bilingual psychosocial professionals from the Syrian community. Based on the results from cognitive interviews, 12 items were excluded, and 1 new item was added (*“feeling unimportant in my family”*), resulting in a 45-item pilot version of the instrument. 18 items were revised, and additional revisions were made in the Arabic translation of the items.

#### Content validity

Six clinical professionals at the RCC rated the relevance of each item of the pilot version of the RPMS in relation to its domain. I-CVIs ranged from 0.80 to 1.00, and S-CVI was calculated to 0.95. Twelve items had an I-CVI lower than 1.00, indicating that at least one rater considered the item to be either somewhat relevant or not relevant. Of these twelve items, only two were rated as being not relevant by two different raters respectively (*“feeling unsafe in my neighborhood”* and *“running into people from my home country who I dislike or don’t trust”*).

#### Pilot testing

The pilot sample (*n* = 41) consisted of 12 women and 28 men (and one person who did not indicate gender), stating Syria, Iraq, Lebanon, Palestine, Kuwait or Yemen as country of origin. Mean age for the participants was 45 years. Based on the settings where they were recruited for the pilot study, it is assumed that all participants had refugee experiences.

The individual inter-item correlation coefficients ranged from − 0.06 to 0.85, mean inter-item correlation for all items was 0.15 (SD = 0.073, range − 0.02-0.29). Items with mean inter-item correlation near zero (*r* < 0.08) indicating lack of common variance (9 items), and individual inter-item correlations close to 1 (*r* > 0.8) indicating item-redundancy (4 items) were identified for further content examination. Also, items displaying a clustering at either side of the response categories (11 items) were deemed to indicate floor (8 items) or ceiling (3 items) effects and were selected for further content examinations.

#### Item selection for the preliminary instrument

An overall evaluation of the pilot version of the instrument, based on usability (results from cognitive interviews), I-CVIs, preliminary statistical characteristics from pilot testing (mean inter-item correlations dispersion, floor and ceiling effects), theoretical considerations regarding item content, and adequate number of items per domain, resulted in a preliminary instrument with 24 items spread across the 7 hypothesized domains.

### Phase 2: construct validity

#### Data collection and sample characteristics

A total of 1215 individuals returned the questionnaire (response rate 30.4%), with younger, unmarried, more recently immigrated individuals and those with lower education level being slightly overrepresented among the non-responders. Sample characteristics are presented in Table 2. For a detailed description of the study and its result, see Tinghög et al. [2].

**Table 2:** Demographic characteristics (%) for respondents and sample frame

Characteristics	Respondents (*n* = 1215)	Sample frame (*n* = 4000)
Gender		
Women	37.2	36.5
Men	62.8	63.5
Age groups (years)		
18–29	23.3	30.8
30–39	32.9	33.7
40–49	24.3	21.0
50–64	19.5	14.6
Marital status		
Unmarried	31.8	40.8
Married	63.5	52.9
Divorced/widow/widower	4.8	6.4
Level of education		
0–9 years	40.2	46.4
> 9 years without a university degree	21.0	22.3
> 12 years with a university degree	38.7	31.5
Year of immigration^a^		
≤ 2011	6.5	10.1
2012	27.5	29.5
2013	66.0	60.4

^a^This variable indicates the year the individual arrived in Sweden and should not be confused with year for being granted residency

#### Confirmatory factor analysis

In the CFA, the outlined 7-factor model of the preliminary 24 item instrument showed a slightly insufficient approximation to fit to the data by a highly significant Satorra–Bentler scaled chi-square (*S-Bχ2* = 872.07; *df* = 231 *p* < 0.001) and a value of CFI marginally lower than 0.95 (CFI = 0.938), SRMR = 0.052, and RMSEA = 0.048 (90% *CI* = 0.045–0.051). After implementing two rounds of re-specifications on the basis of Modification Indices, consisting of sequential addition of two error term covariances to the model (*“difficulties understanding how ordinary life activities in Sweden work”* and *“being unable to buy necessities”*, and *“discrimination by Swedish authorities”* and *“discrimination in school or at work”*, respectively), the results still failed to show fit to the data (*S-Bχ2* = 813.10; *df* = 229 *p* < 0.001, CFI = 0.941, SRMR = 0.052, and RMSEA = 0.046 (90% *CI* = 0.042–0.049). Further inspection of the Modification Indices as well as the residual variances in the model revealed several potential misspecifications, including items with poor factorial loading and substantive cross loadings. As no further theoretically justifiable modifications were possible, and since re-specification of the model based solely on Modification Indices would lead us into the data-driven, exploratory realm [56], we followed recommendations by Asparouhov and Muthén and proceeded with EFA within ESEM [57].

#### Exploratory factor analysis

EFA within ESEM with Geomin rotation was performed in 4 iterations resulting in the omitting of three items, *“being unable to buy necessities”* (substantive cross loadings), *“fear of being sent home”* (poor overall factor loading < 0.2), and *“running into people from my home country who I dislike or don’t trust”* (cross loading and poor factor loading < 0.2). An 8-factorial model of the remaining 21 item instrument resulted in a non-significant Satorra–Bentler scaled chi-square (*S-Bχ2* = 81.70; *df* = 70 *p* = 0.160) but contained one factor with no items with significant factor loadings and was hence subsequently discarded. A 7-factorial solution provided excellent fit indices (CFI = 0.992, SRMR = 0.011, and RMSEA = 0.027, 90% *CI* = 0.020–0.033), although the Satorra–Bentler scaled chi-square was still significant (*S-Bχ2* = 156.10; *df* = 84 *p* < 0.001). Given the overall sensitivity of the chi-square statistics to large sample sizes, and the interpretability of the 7-factorial solution, this model was selected. Items, factors, and corresponding factorial loadings are displayed in Additional file 1, whereas factor correlations are shown in Additional file 2. Descriptive statistics for the RPMS are shown in Additional file 3.

#### Internal consistency

Cronbach’s alpha for the seven factors ranged between 0.74 and 0.87 and indicated acceptable to good internal consistency. For the total scale, Cronbach’s alpha was 0.86. Cronbach’s alpha for all seven factors and for the total scale are shown in Table 3.

**Table 3:** Cronbach’s alpha for the seven factors of post-migration stress and for total scale, and Pearson correlations between factors and total scale, and anxiety, depression, PTSD, and mental wellbeing, respectively

Factor	Cronbach’s alpha	Anxiety	Depression	PTSD	Mental wellbeing
Perceived discrimination	0.82	0.27^b^	0.28^b^	0.24^b^	−0.24^b^
Lack of host country specific competences	0.80	0.25^b^	0.34^b^	0.36^b^	−0.31^b^
Material and economic strain	0.84	0.44^b^	0.54^b^	0.51^b^	−0.49^b^
Loss of home country	0.87	0.23^b^	0.29^b^	0.30^b^	−0.26^b^
Family and home country concerns	0.74	0.15^b^	0.18^b^	0.19^b^	−0.15^b^
Social strain	0.81	0.39^b^	0.52^b^	0.47^b^	−0.44^b^
Family conflicts	0.82	0.29^b^	0.38^b^	0.33^b^	−0.34^b^
RPMS total	0.86	0.44^b^	0.54^b^	0.49^b^	−0.47^b^

^a^ The correlation is significant at the 0.05 level (2-tailed)

^b^ The correlation is significant at the 0.01 level (2-tailed)

#### Zero-order correlations

All post-migration stress factors correlated significantly with HSCL-25 anxiety and depression scales, and with HTQ. Furthermore, all post-migration stress factors displayed significant negative correlations with WHO-5 mental wellbeing scores. The total scale showed a similar pattern of correlations. *Material and economic strain* (*r* between 0.44 and 0.54) and *social strain* (*r* between 0.39 and 0.52) contained a pattern of moderate coefficients with all measures of mental ill health and wellbeing, whereas *family and home country concerns* contained the weakest pattern of coefficients (*r* between 0.15 and 0.19). Correlations are shown in Table 3.

## Discussion

Despite the growing body of evidence for the impact of post-resettlement factors on the mental health of refugees, a validated instrument for assessment of post-migration stress among refugees is still lacking. The aim of this study was to develop and validate a concise, multidimensional self-assessment instrument for evaluation of post-migration stress among refugees, based on a theoretical conceptualization and empirical evidence of the construct of post-migration stress.

A systematic development process resulted in a 21-item instrument, the Refugee Post-Migration Stress Scale (RPMS), assessing stress related to post-resettlement experiences and life conditions across seven domains. Our findings suggest that the RPMS is a usable and valid instrument for assessing post-migration stress among refugees. The results reveal that the empirical data supports the conceptualized multi-domain model of post-migration stress. Furthermore, the associations between different facets of post-migration stress and mental ill health among refugees found in this study are in line with previous research [4, 7, 58].

Associations between the domains of post-migration stress in RPMS and the included measures of mental health were generally moderate, indicating that what the RPMS assesses is different from anxiety, depression, PTSD and low mental wellbeing. With a moderate association found between PTSD and post-migration stress, it needs to be pointed out that while there may be similarities between traumatic experiences and experiences of post-migratory stress, there are also important differences, mainly that post-migration stress is seen as the subjective appraisal of *reoccurring or persistent post-resettlement related living conditions* as distressing. Whereas HTQ evaluates symptoms of post-traumatic stress, the RPMS assess experiences of post-migration stress. As such, although these constructs may be confounded, our theoretical clarification is an attempt to articulate conceptual differences between post-migratory stress and psychological distress and mental ill health experienced by refugees.

Although the CFA did fail to show adequate fit between our theorized model and the observed data, the follow-up EFA showed that the factorial structure corresponding to the theorized domains of post-migration stress was appropriate. The indicated lack of support for the initial multi-domain model in the CFA appears to be due to problematic content on item level, that is, several items did not adequately reflect the hypothesized latent domains of post-migration stress. As one item was removed from the family and home country concerns factor, it now contains only two items. Despite the lack of robustness for two-item factors, we found it theoretically motivated to retain the factor in the final version of the RPMS, as the removal of it would mean that important aspects of post-migration stress would not be covered by the RPMS. However, the factor will optimally need to be further developed in future studies, with the aim at reaching at least three items for the factor.

Based on both theory and item content, it could be argued that the 7-domain structure of post-migration stress could be further split into two broad, content-wise different categories. In fact, our post hoc analyses (results not shown) of examining the factorial structure for four domains relating to *host society stress*, and three domains relating to *family and home country stress* separately, indicate excellent fit for these two categories. Whether these findings reflect a hierarchical structure of post-migration stress, meaning that these two categories form a single higher-order latent construct, or a bi-factorial model [59] of post-migration stress, could be investigated in future studies.

In the same line, the domains more pronouncedly related to *host society stress* (*perceived discrimination, lack of host country specific competences, material and economic strain* and *social strain)*, displayed a pronounced pattern of correlations with all mental health outcomes. This is not surprising, as the link between poor socioeconomic conditions and mental ill health has been shown in previous research on refugees [12], non-refugee migrants [15] and non-migrants [60]. Although harsh socioeconomic conditions are obviously not refugee-specific, this association is important since refugees are at greater risk of living under poor conditions compared to the general population [61], hence being at risk for developing mental ill health relating to these conditions [12, 62, 63]. The association between the *host society stress* domains and mental ill health also points to the possibilities to reduce the risk for developing mental ill health among refugees by interventions targeting discrimination, unemployment, and social isolation.

For the domains relating to *family and home country stress* (*loss of home country, family and home country concerns,* and *family conflicts),* the correlations with mental health outcomes were weaker. While the negative impact of forced family separation on the mental health of refugees has indeed been shown in several studies [9, 13], the *family and home country stress* domains also contain items representing more general human conditions relating to uprooting, migration and resettlement, such as longing for the lost home country and being far from family members and relatives. Certainly, these experiences would be somewhat distressing to most, but not necessarily would they be associated with mental ill health. Moreover, it is possible that the consequences of *home country and family stress* on the mental health of refugees become evident only after a certain time in the resettlement country, and hence would not necessarily be present in our sample of newly resettled refugees.

Our findings that mental ill health among recently resettled refugees mainly appears to be associated with host society stress is in line with previous research [12, 15, 17]. Further investigations on how these different domains of post-migration stress are associated with one another are needed for a more in-depth understanding of the complex patterns of post-resettlement experiences and living conditions faced by refugees. Future studies investigating the psychometric proprieties of the RPMS should, furthermore, include Item Response Theory (IRT) analysis of each subscale in order to enable person estimates to be calculated from item responses [64], as well as cross-validation and invariance testing of the factorial structure in independent samples.

### Strengths and limitations

In the current study we found support for our theorized model of refugee post-migration stress. The empirical data used in the validation of the RPMS comes from a large random sample drawn from a known sample frame with a satisfactory response rate. Although some groups were slightly overrepresented among the non-responders, the sample can still be viewed as adequately representative [2]. Hence, the RPMS appears to be an appropriate and valid instrument for assessing post-migration stress, at least among refugees from Syria.

However, the fact that our sample consisted solely of refugees from Syria that had recently resettled, is a limitation of this study. Also, since no testing of measurement invariance has been performed, the stability of the RPMS across different subgroups (e.g., gender, age, time spent in host country) as well as across different refugee groups, including asylum seekers, remains unknown. Thus, the RMPS needs to be tested and validated among other refugee populations in different high-income counties to ensure its generalizability.

A possible limitation of this study is that there may be domains missing from the RPMS that would be relevant to specific refugee groups living in different contexts, and that could be equally important as any of the constructs included in the RPMS. Such domains could favorably be added to the RPMS to more adequately capture the experiences of post-resettlement stress in these specific refugee populations.

In the present study we chose to follow up the CFA with an EFA within ESEM with Geomin rotation to achieve a robust model, while at the same time ensuring that the model was closely in line with our a priori reasoning. As this meant moving from confirmatory to exploratory work, it could be argued that we went a bit too far in this quest. An alternative modeling approach would have been ESEM with a targeted rotation, a type of modeling strategy that can be perceived as following a stricter confirmatory approach [54]. We believe that future studies of RPMS should not only be done on new independent samples, but alternative modeling strategies should also be explored to gain increased insights in the psychometric properties of the RPMS.

Alpha values for the RPMS may have been influenced by multidimensionality and local dependence, as a full construct validation could not be performed due to the lack of relevant information produced in the ESEM analyses [65]. This is of particular risk when calculating Cronbach’s alpha for the entire scale. Future studies on the RPMS should also include testing the dimensionality of items.

It needs to be pointed out that the correlational analyses in this study were conducted for validation purposes, and hence potential confounders were not controlled for. Therefore, associations between the different domains of post-migration stress the mental health outcomes should be interpreted cautiously. Also, because of the cross-sectional study design, it is not possible to make inferences on causality between post-migration stress and mental health. In future research, longitudinal studies are needed to establish the potential causal mechanisms between post-migration stress and mental health among refugees.

## Conclusions

The RPMS, developed in the current study, appears to be a valid and usable instrument for assessing post-migration stress among refugees from Syria with residence permit in high-income country settings. Post-migration stress primarily relating to social and economic factors seems to be associated with mental ill health among refugees, which points out the need for interventions targeting discrimination, unemployment, and social isolation.

## Supplementary information

**Additional file 1.** Items, factors, and corresponding factorial loadings for the 7-factorial solution of post-migration stress in the exploratory factor analysis (EFA).

**Additional file 2.** Geomin correlations for the seven factors of the Refugee Post-Migration Stress Scale (RPMS).

**Additional file 3.** Descriptive statistics for the Refugee Post-Migration Stress Scale (RPMS).

## Data Availability

The statistical code is available from the corresponding author. Under Swedish law and ethical approval, individual level data of this kind cannot be publicly available. Individual level data can be made available on reasonable request as long as it is in line with Swedish law and ethical approvals.

## Abbreviations

CFA: Confirmatory factor analysis
CFI: Comparative fit index
CI: Confidence interval
df: Degrees of freedom
EFA: Exploratory factor analysis
ESEM: Exploratory structural equation modelling
HSCL-25: Hopkins Symptom Checklist-25
I-CVI: Item level content validity index
IRT: Item Response Theory
MLR: Robust maximum likelihood estimation
PTSD: Post-traumatic stress disorder
RCC: The Swedish Red Cross Treatment Center for Persons Affected by War and Torture
RMSEA: Root mean square error of approximation
RPMS: Refugee Post-Migration Stress Scale
S-Bχ2: Satorra-Bentler scaled chi-square
S-CVI: Scale level content validity index
SRMR: Standardized root mean square residual
WHO-5: WHO-5 Well-being Index
