# Supplementary material for: The refugee post-migration stress scale (RPMS) – development and validation among refugees from Syria recently resettled in Sweden
Source: Confl Health. 2020 Jan 6;14:2. doi: 10.1186/s13031-019-0246-5 (PMC6945710; doi:10.1186/s13031-019-0246-5)
Supplement: Supplementary file 1 — Additional file 1. Items, factors, and corresponding factorial loadings for the 7-factorial solution of post-migration stress in the exploratory factor analysis (EFA). [file 13031_2019_246_MOESM1_ESM.docx]

**Additional file 1** Items, factors, and corresponding factorial loadings for the 7-factorial solution of post-migration stress in the exploratory factor analysis (EFA). The strongest factorial loadings for each factor appear bold in the table.

| Item | Factors  Perceived discrimination | Lack of host country specific competences | Material and economic strain | Loss of home country | Family and home country concerns | Social strain | Family conflicts |
| --- | --- | --- | --- | --- | --- | --- | --- |
| Discrimination by Swedish authorities | **0.638*** | 0.035 | 0.001 | -0.033 | 0.010 | 0.037 | 0.009 |
| Discrimination in school or at work | **0.701*** | -0.064* | 0.067 | 0.008 | -0.018 | -0.013 | -0.012 |
| Feeling disrespected due to my national background | **0.796*** | -0.030 | -0.018 | 0.004 | -0.012 | 0.040 | 0.016 |
| People making racist remarks towards me | **0.756*** | 0.037 | -0.019 | 0.022 | 0.010 | -0.030 | -0.004 |
| Bothering difficulties communicating in Swedish | 0.082* | **0.628*** | 0.025 | 0.033 | 0.005 | 0.108* | -0.037 |
| Difficulties understanding how ordinary life activities in Sweden work (shopping, buying tickets, traveling, etc.) | -0.010 | **0.771*** | -0.030 | -0.023 | 0.030 | 0.019 | 0.009 |
| Difficulties understanding documents and forms from authorities | -0.054* | **0.827*** | 0.042 | 0.013 | -0.025 | -0.027 | 0.022 |
| Worry about unstable financial situation | 0.041** | 0.072* | **0.818*** | 0.032 | -0.001 | -0.046* | 0.003 |
| Frustration for not being able to support myself financially | -0.012 | 0.012 | **0.840*** | 0.005 | -0.020 | 0.035 | 0.014 |
| Worry about debts | -0.021 | -0.055* | **0.690*** | -0.038 | 0.124* | 0.043 | 0.001 |
| Missing my social life from back home | 0.042* | 0.009 | 0.047* | **0.810*** | 0.021 | 0.005 | -0.004 |
| Longing for my home country | -0.013 | 0.032 | -0.084* | **0.886*** | 0.026 | -0.021 | -0.015 |
| Missing activities that I used to do before coming to Sweden | -0.015 | -0.042 | 0.044 | **0.760*** | -0.032 | 0.086* | 0.027 |
| Worry about family members that I am separated from | -0.004 | 0.035 | 0.001 | 0.190* | **0.628*** | -0.015 | 0.006 |
| Feeling sad because I am not reunited with family members | -0.004 | -0.024 | 0.008 | -0.012 | **0.889*** | 0.014 | -0.008 |
| Feeling excluded or isolated in the Swedish society | 0.143* | 0.111* | 0.017 | -0.011 | 0.131* | **0.543*** | 0.055 |
| Frustration due to loss of status in the Swedish society | -0.023 | -0.030 | -0.008 | 0.004 | -0.013 | **0.994*** | -0.011 |
| Frustration because I am not able to make use of my competences in Sweden | 0.010 | 0.039 | 0.256* | 0.107* | -0.020 | **0.493*** | -0.008 |
| Distressing conflicts in my family | 0.054 | 0.030 | 0.030 | -0.025 | 0.020 | 0.081* | **0.606*** |
| Feeling disrespected in my family | 0.009 | -0.016 | -0.012 | 0.013 | 0.001 | -0.036* | **0.975*** |
| Feeling unimportant in my family | -0.040* | 0.001 | 0.004 | 0.006 | -0.015 | 0.012 | **0.841*** |

* Significant at 0.05 level.
