# Supplementary material for: The refugee post-migration stress scale (RPMS) – development and validation among refugees from Syria recently resettled in Sweden
Source: Confl Health. 2020 Jan 6;14:2. doi: 10.1186/s13031-019-0246-5 (PMC6945710; doi:10.1186/s13031-019-0246-5)
Supplement: Supplementary file 2 — Additional file 2. Geomin correlations for the seven factors of the Refugee Post-Migration Stress Scale (RPMS). [file 13031_2019_246_MOESM2_ESM.docx]

**Additional file 2** Geomin correlations for the seven factors of the Refugee Post-Migration Stress Scale (RPMS).

|  | Perceived discrimination | Lack of host country specific competences | Material and economic strain | Loss of home country | Family and home country concerns | Social strain | Family conflicts |
| --- | --- | --- | --- | --- | --- | --- | --- |
| Perceived discrimination |  |  |  |  |  |  |  |
| Lack of host country specific competences | 0.063 |  |  |  |  |  |  |
| Material and economic strain | 0.217* | 0.434* |  |  |  |  |  |
| Loss of home country | 0.122* | 0.350* | 0.361* |  |  |  |  |
| Family and home country concerns | 0.173* | 0.140* | 0.256* | 0.032 |  |  |  |
| Social strain | 0.394* | 0.400* | 0.544* | 0.372* | 0.258* |  |  |
| Family conflicts | 0.068 | 0.213* | 0.193* | 0.224* | 0.069* | 0.193* |  |

* Significant at 5 % level.
