# Supplementary material for: The refugee post-migration stress scale (RPMS) – development and validation among refugees from Syria recently resettled in Sweden
Source: Confl Health. 2020 Jan 6;14:2. doi: 10.1186/s13031-019-0246-5 (PMC6945710; doi:10.1186/s13031-019-0246-5)
Supplement: Supplementary file 3 — Additional file 3. Descriptive statistics for the Refugee Post-Migration Stress Scale (RPMS). [file 13031_2019_246_MOESM3_ESM.docx]

**Additional file 3** Descriptive statistics for the Refugee Post-Migration Stress Scale (RPMS).

| Item | Minimum | Maximum | Mean | Standard  deviation |
| --- | --- | --- | --- | --- |
| Discrimination by Swedish authorities | 1 | 5 | 1.53 | 0.901 |
| Discrimination in school or at work | 1 | 5 | 1.75 | 1.034 |
| Feeling disrespected due to my national background | 1 | 5 | 1.61 | 0.934 |
| People making racist remarks towards me | 1 | 5 | 1.66 | 0.919 |
| Bothering difficulties communicating in Swedish | 1 | 5 | 3.09 | 1.386 |
| Difficulties understanding how ordinary life activities in Sweden work (shopping, buying tickets, traveling, etc.) | 1 | 5 | 2.00 | 1.178 |
| Difficulties understanding documents and forms from authorities | 1 | 5 | 2.81 | 1.312 |
| Worry about unstable financial situation | 1 | 5 | 3.22 | 1.378 |
| Frustration for not being able to support myself financially | 1 | 5 | 2.74 | 1.460 |
| Worry about debts | 1 | 5 | 2.52 | 1.513 |
| Missing my social life from back home | 1 | 5 | 3.93 | 1.247 |
| Longing for my home country | 1 | 5 | 4.04 | 1.186 |
| Missing activities that I used to do before coming to Sweden | 1 | 5 | 3.75 | 1.304 |
| Worry about family members that I am separated from | 1 | 5 | 3.90 | 1.492 |
| Feeling sad because I am not reunited with family members | 1 | 5 | 3.13 | 1.796 |
| Feeling excluded or isolated in the Swedish society | 1 | 5 | 2.39 | 1.304 |
| Frustration due to loss of status in the Swedish society | 1 | 5 | 2.43 | 1.383 |
| Frustration because I am not able to make use of my competences in Sweden | 1 | 5 | 3.06 | 1.498 |
| Distressing conflicts in my family | 1 | 5 | 1.60 | 0.987 |
| Feeling disrespected in my family | 1 | 5 | 1.25 | 0.670 |
| Feeling unimportant in my family | 1 | 5 | 1.24 | 0.705 |
